# Sarcopenia accompanied by systemic inflammation can predict clinical outcomes in patients with head and neck cancer undergoing curative therapy

**DOI:** 10.3389/fonc.2024.1378762

**Published:** 2024-03-14

**Authors:** Ken Kasahara, Takeyuki Kono, Yoichiro Sato, Masafumi Ueno, Hirotaka So, Yoshimitsu Fuse, Seiichi Shinden, Hiroyuki Ozawa

**Affiliations:** ^1^ Department of Otolaryngology and Head and Neck Surgery, Keio University School of Medicine, Shinjuku-ku, Tokyo, Japan; ^2^ Division of Otolaryngology and Head and Neck Surgery, Saiseikai Utsunomiya Hospital, Utsunomiya-Shi, Tochigi, Japan

**Keywords:** sarcopenia, nutrition, systemic inflammation, overall survival, progression-free survival, prognostic marker, head and neck cancer

## Abstract

**Objectives:**

Evaluation of sarcopenia accompanied by systemic inflammation status is a more beneficial prognostic marker than sarcopenia alone in various cancers. However, few studies have focused on this combination in patients with head and neck squamous cell cancer (HNSCC). In this study, we investigated how the combination of sarcopenia and systemic inflammation could affect survival in patients with HNSCC. Moreover, we explored which systemic inflammation markers could be better prognostic indicators when accompanied by sarcopenia.

**Materials and methods:**

We retrospectively reviewed the medical records of patients with HNSCC treated between 2012 and 2016. Sarcopenia was defined by the skeletal muscle area measured on a computed tomography image slice at the level of the third cervical vertebra. The neutrophil/lymphocyte, platelet/lymphocyte, and lymphocyte/monocyte ratios (NLR, PLR, and LMR, respectively) were used as systemic inflammation markers that were combined with sarcopenia to evaluate prognosis.

**Results:**

A total of 100 patients were enrolled, and 71 patients were considered sarcopenia. Patients with sarcopenia had significantly lower LMR and higher NLR and PLR. They also showed worse overall survival (OS) and progression-free survival (PFS). The comparative assessment of multiple combination patterns of sarcopenia and systemic inflammation indices proved that sarcopenia plus LMR considered as most reliable indicator for prognosis in HNSCC patients. Sarcopenia plus low LMR was a significantly poor prognostic factor both for OS and PFS with greater HR values than sarcopenia alone.

**Conclusions:**

The combination of sarcopenia and LMR was considered the most sensitive prognostic factor in patients with HNSCC, suggesting it might be beneficial for identifying poor outcome risks.

## Introduction

Numerous studies have described that malnutrition and systemic inflammation closely correlate to poor outcomes in patients with malignant tumors (1, 2). Sarcopenia, a loss of skeletal muscle mass (SMM), muscle strength, or loss of physical function, is among malnutrition status indicators associated with poor outcomes such as physical disability, poor life quality (3), and worse prognosis in patients with several cancers. In particular, patients with head and neck squamous cell cancer (HNSCC) are at risk for sarcopenia as the tumor site might cause dysphagia and difficulties in swallowing, and a recent study described that sarcopenia increased chemotherapeutic toxicity and is an independent risk factor for poor overall survival (OS) in patients with HNSCC (4).

Meanwhile, systemic inflammation also plays an important role in cancer patients. The neutrophil/lymphocyte, platelet/lymphocyte, and lymphocyte/monocyte ratio (NLR, PLR, and LMR, respectively) are widely used as systemic inflammation markers. Previous studies have demonstrated that higher NLR and PLR as well as lower LMR levels are associated with poor outcomes in patients with cancer (5–8).

Sarcopenia closely correlates to systemic inflammation, which causes muscle degeneration, leading to sarcopenia in patients with cancer (9, 10). Considering these aspects, recent studies described that sarcopenia with systemic inflammation is central to determining survival in various cancers. However, few studies have examined how sarcopenia accompanied by systemic inflammation could affect the prognosis of patients with HNSCC. Therefore, in this study, we investigated whether the combined evaluation of sarcopenia and systemic inflammation could serve as a reliable prognostic marker in patients with HNSCC who underwent curative therapy by comparing several combination patterns including sarcopenia with NLR, PLR, and LMR.

## Materials and methods

### Study design and patients

In this retrospective study, we included a total of 100 patients with HNSCC who had received initial treatment for primary HNSCC such as cancer of the oropharynx, hypopharynx, and larynx between February 2012 and March 2016. Exclusion criteria involved patients with missing data and undergoing palliative therapy only. All clinical data were collected using electronic medical records. This study was approved by the appropriate institutional research ethics committee (reference numbers: 2019-29) and was conducted according to the tenets of the Declaration of Helsinki. The requirement for informed consent was waived owing to the retrospective nature of the analysis.

### Treatment protocol

Patients were treated with surgery, radiotherapy (RT) alone, and concurrent chemoradiotherapy (CCRT) considering various factors such as age, stage of disease, risk factors, performance status, and comorbidities. The listed treatments were initiated according to the guidelines of the National Comprehensive Cancer Network. Briefly, T1 and T2 cases prefer RT alone (total of 60–66 Gy) or transoral surgery, while T3 and T4 cases were administered CCRT (Cisplatin; 80 mg/m^2^, infused on days 1, 22, and 43, RT; a total of 66 Gy) or surgery (total laryngectomy or pharyngolaryngectomy) based on several factors of the patients. In advanced cases, surgery was preferred.

### Measurement of skeletal muscle cross-sectional area and sarcopenia definition

**Figure 1:**
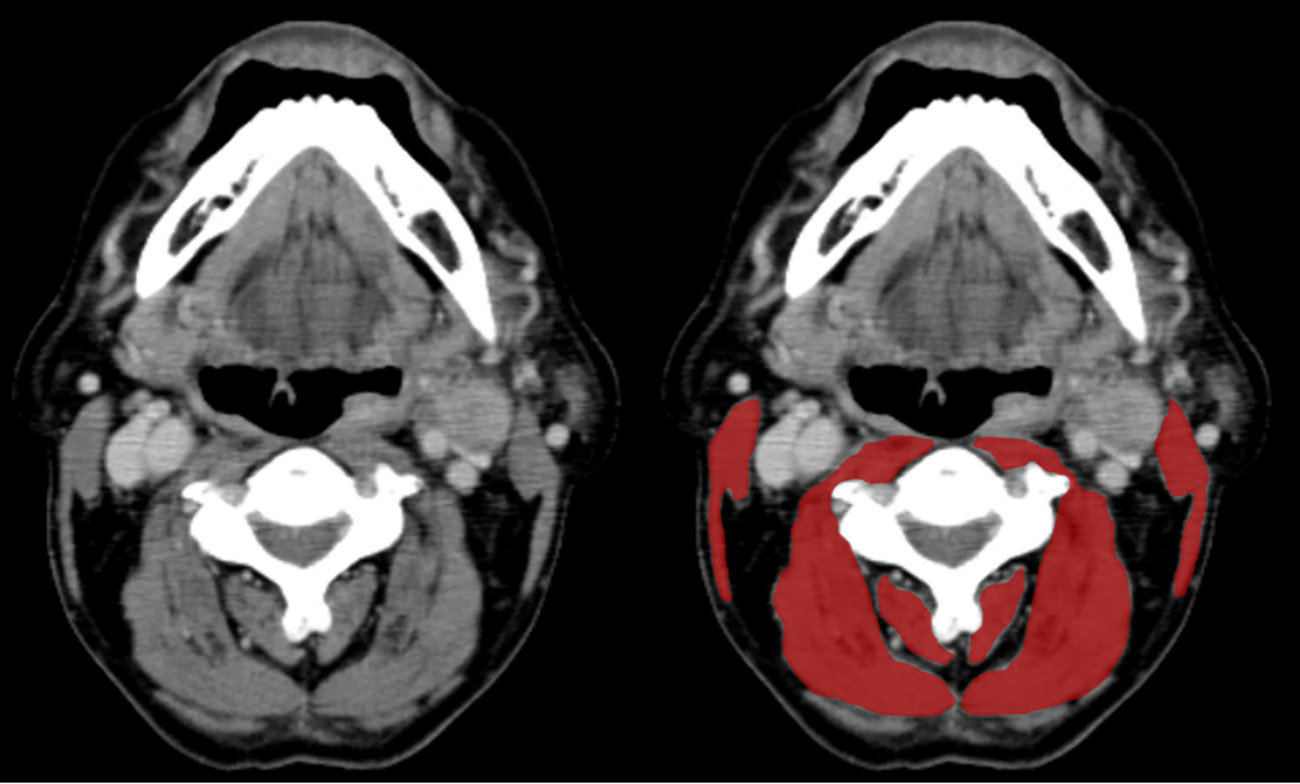
Paravertebral and sternocleidomastoid muscle area measurement at the level of the C3 vertebra. In the left axial CT slide, the muscle tissue is unsegmented. The right CT slide shows both the paravertebral and sternocleidomastoid segmented muscles in red.


In all 100 cases, cervical computed tomography (CT) imaging was obtained before the treatment. According to a previously described method by Swarz et al. (11), SMM was determined in each patient. Briefly, a single axial CT slide at level C3, displaying the entire vertebral arc, was selected first, when the C3 vertebra was scrolled from a caudal to a cephalic direction. The paravertebral muscle and both sternocleidomastoid muscle segments were highlighted in red and traced manually using the ImageJ software (**Figure 1**). We calculated the sum of the delineated areas of both the paravertebral and sternocleidomastoid muscles at the level of C3 vertebrae, defined as the cross-sectional muscle area (CSA) at level C3. Next, we estimated CSA at level L3 using the prediction method previously described by Swartz et al. (11). The estimated CSA at level L3 was normalized for the height by dividing it by the squared height, defined as the lumber skeletal muscle index (LSMI, cm^2^/m^2^).

$$\begin{array}{l} \text{CSA at level L}3\;({\text{cm}}^{2})=27.304+1.363*\text{CSA at level C}3\;({\text{cm}}^{2})-0.671*\text{Age}+0.640*\\ \text{weight}\left(\text{kg}\right)+26.442*\text{Sex }\left(1\text{ for female},\;2\text{ for male}\right) \end{array}$$

$$\text{LSMI}\;\left({\text{cm}}^{2}/{\text{m}}^{2}\right)=\text{CSA at level L}3\;\left({\text{cm}}^{2}\right)/\text{length}\;\left({\text{m}}^{2}\right)$$

Sarcopenia is characterized by an LSMI below 43.2cm^2^/m^2^ (12) according to the international consensus. In our study, although only low LSMI was used for sarcopenia definition, albumin and BMI were also collected as indicators reflecting nutritional status. Based on institutional criteria, the cut-off values of albumin and BMI were set at 3.5 and 18.5, respectively.

### Systemic inflammation markers

The blood cell counts of the patients were measured within one week of treatment administration. NLR, PLR, and LMR were calculated by dividing the neutrophil count by the lymphocyte count, the platelet count by the lymphocyte count, and the lymphocyte count by the monocyte count, respectively. Low values of LMR and high values of NLR and PLR suggest high inflammatory status. We developed Receiver Operating Characteristic (ROC) curves for the NLR, PLR, and LMR using OS as the primary endpoint. NLR ≥ 2.180 was defined as high with an area under the curve (AUC), sensitivity, and specificity of 0.62, 60.0%, and 62.0%, respectively. A high PLR was defined as ≥ 112.8 with an AUC, sensitivity, and specificity of 0.59, 42.0%, and 84.0%, respectively. A lower LMR was defined as ≤ 4.118 with an AUC, sensitivity, and specificity of 0.62, 68.0%, and 58.0%, respectively.

### Statistical analysis

Continuous variables are shown as the median (or mean) and range, while we presented categorical variables as frequencies. For comparisons between groups, we analyzed continuous data using the Mann–Whitney U test, while we performed categorical data analysis using Fisher’s exact test or the Chi-square test. We defined OS as the time from diagnosis until the last follow-up or death from any cause. We defined progression-free survival (PFS) as the time from diagnosis until the detection of the first detection of disease progression, the last follow-up, or death from any cause. We also compared how potential risk factors (age, sex, primary site, T or N classification, treatment type, anemia, BMI, sarcopenia, NLR, PLR, or LMR) could affect OS and PFS using the log-rank test and analyzed by generating Kaplan–Meier survival curves. We used Cox hazard regression analysis to perform multivariable analysis on the variables with P-values of *p* < 0.05 in the univariate analysis and clinically important OS and PFS predictors. HRs and their corresponding 95% confidence intervals are presented. We compared the combined prognostic factor of sarcopenia and systemic inflammation markers (NLR, PLR, and LMR) according to the ROC curve and also calculated the AUC. All statistical analyses were performed using EZR (Saitama Medical Center, Jichi Medical University, Saitama, Japan), a graphical user interface for R (The R Foundation for Statistical Computing, Vienna, Austria). More precisely, the referred interface is a modified version of R commander designed to add statistical functions that are frequently used in biostatistics. P < 0.05 was considered statistically significant.

## Results

### Patient characteristics

In a total of 100 patients, 94 were men and the median age at diagnosis was 69 years (range, 39-92 years). Of these, 12, 24, and 64 patients suffered from oropharyngeal, hypopharyngeal, and laryngeal cancer, respectively. The patients were divided into non-sarcopenia and sarcopenia groups, according to the LSMI cut-off described in the Material and Methods section. **Table 1** presents the patient characteristics in the non-sarcopenia (n = 29, 29%) and sarcopenia (n = 71, 71%) groups. Patients in the sarcopenia group were older, at a more advanced T- and TNM stage, and displayed lower BMI compared to those in the non-sarcopenia group while values of albumin did not show significant differences. Concerning the inflammatory markers, NLR and PLR were significantly higher in the sarcopenia than in the non-sarcopenia group (*p* = 0.021 and 0.031, respectively) and LMR was significantly lower in the sarcopenia than in the non-sarcopenia group (*p* = 0.040).

**Table 1:** Patient and disease characteristics in sarcopenia and non-sarcopenia groups.

Characteristics		Total		Non-sarcopenia		Sarcopenia		*p*-value
		N = 100	%	N = 29	%	N = 71	%	
Age	Mean ± SD	68 ± 9		65 ± 8		69 ± 10		0.031
Sex	Male	94	94	25	86.2	69	97.2	0.057
	Female	6	6	4	13.8	2	2.8	
Primary site	Oropharynx	12	12	1	3.4	11	15.5	0.012
	Hypopharynx	24	24	3	10.3	21	29.6	
	larynx	64	64	25	86.2	39	54.9	
T classification	1–2	71	71	25	86.2	45	64.8	0.001
	3–4	29	29	4	13.8	25	35.2	
N classification	0	70	70	24	82.8	46	64.8	0.094
	1–3	30	30	5	17.2	25	35.2	
TNM stage	I–II	57	57	24	82.7	33	46.5	< 0.001
	III–IV	43	43	5	17.2	38	53.5	
Treatment	Surgery	22	22	1	3.5	21	29.6	0.009
	RT alone	26	26	9	31.0	17	23.9	
	CCRT	52	52	19	65.5	33	46.5	
BMI (kg/m^2^)	Mean ± SD	22.3 ± 3.1		24.3 ± 3.0		21.5 ± 2.8		< 0.001
Albumin (g/l)	Mean ± SD	4.0 ± 0.5		4.1 ± 0.4		4.0 ± 0.6		0.227
NLR	Mean ± SD	2.7 ± 1.9		1.9 ± 1.0		3.0 ± 2.1		0.021
PLR	Mean ± SD	157.8 ± 78.4		137.8 ± 67.4		166.0 ± 81.5		0.040
LMR	Mean ± SD	4.7 ± 2.2		5.4 ± 2.1		4.5 ± 2.2		0.031

BMI (body mass index); NLR (neutrophil/lymphocyte ratio); PLR (platelet/lymphocyte ratio); LMR (lymphocyte/monocyte ratio); SD (standard deviation); RT (radiotherapy); CCRT (concurrent chemoradiotherapy).

### Survival and prognostic factor analysis

**Figure 2:**
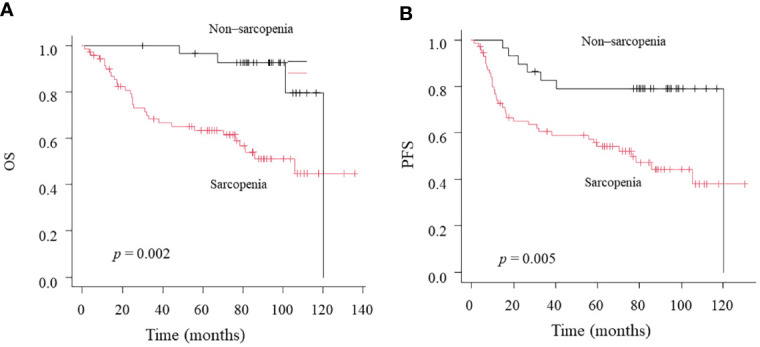
Kaplan-Meier curves comparing OS **(A)** and PFS **(B)** between the Non-sarcopenia and Sarcopenia.

Twenty-eight patients died over a median follow-up duration of 78 months (range, 1–138 months). The 3-year OS and PFS rates among all 100 patients were 79 and 77%, respectively. Our univariate analysis revealed that NLR was associated with OS, but not with PFS (**Table 2**). Moreover, the univariate analysis showed that T classification, sarcopenia, and LMR were associated both with OS and PFS. Multivariate analysis using factors that showed significant differences in univariate analysis revealed that only sarcopenia was a significant predictor of both OS and PFS. According to the Kaplan–Meier analysis, the patients with sarcopenia had poorer OS (log-rank test: *p* = 0.002; **Figure 2A**) and PFS (log-rank test: *p* = 0.005; **Figure 2B**) than those with non-sarcopenia.

**Table 2:** Prognostic factors for OS and PFS in patients with HNSCC.

variables		OS				PFS			
		Univariate		Multivariate		Univariate		Multivariate	
		HR (95% CI)	*p*-value	HR (95% CI)	*p*-value	HR (95% CI)	*p-*value	HR (95% CI)	*p-*value
**Age**	**<65**	1				1			
	**≧65**	1.68 (0.78–3.62)	0.184			1.17 (0.61–2.22)	0.635		
**Sex**	**Female**	1				1			
	**Male**	1.90 (0.25–13.92)	0.528			2.51 (0.34–18.28)	0.364		
**Primary site**	**Oropharynx**	1				1			
	**Hypopharynx**	2.08 (0.57–7.57)	0.268			2.90 (0.80–10.47)	0.105		
	**Larynx**	1.45 (0.43–4.91)	0.546			2.13 (0.64–7.09)	0.217		
**T classification**	**1–2**	1		1		1		1	
	**3–4**	2.42 (1.22–4.83)	0.012	1.38 (0.64–2.99)	0.414	2.71 (1.47–5.02)	0.001	1.36 (0.62–2.98)	0.443
**N classification**	**0**	1				1			
	**1–3**	1.25 (0.61–2.58)	0.548			1.47 (0.78–2.76)	0.236		
**Treatment**	**Surgery**	1				1			
	**Non–surgery**	1.034 (0.46–2.32)	0.936			1.129 (0.54–2.37)	0.747		
**Albumin (g/l)**	**≧3.5**	1				1		1	
	**< 3.5**	1.68 (0.85–3.31)	0.135			1.84 (1.01–3.36)	0.046	1.23 (0.59–2.57)	0.589
**BMI (kg/m^2^)**	**≧18.5**	1				1			
	**< 18.5**	0.71 (0.22–2.34)	0.576			0.76 (0.27–2.14)	0.610		
**Sarcopenia**	**NonSarcopenia**	1		1		1		1	
	**Sarcopenia**	4.57 (1.61–13.03)	0.004	3.35 (1.12–10.03)	0.030	3.00 (1.33–6.77)	0.008	3.30 (1.11–9.83)	0.032
**NLR**	**Normal**	1		1		1			
	**High**	2.06 (1.04–4.07)	0.038	1.21 (0.54–2.75)	0.641	1.50 (0.82–2.72)	0.187		
**PLR**	**Normal**	1				1			
	**High**	1.42 (0.66–3.05)	0.376			0.95 (0.50–1.80)	0.876		
**LMR**	**Normal**	1		1		1		1	
	**Low**	2.92 (1.44–5.93)	0.003	1.96 (0.87–4.41)	0.105	2.03 (1.11–3.72)	0.022	2.09 (0.99–4.41)	0.053

OS (overall survival); PFS (progression-free survival); HR (hazard ratio); CI (confidence interval); BMI (body mass index); NLR (neutrophil/lymphocyte ratio); PLR (platelet/lymphocyte ratio); LMR (lymphocyte/monocyte ratio).

### ROC analysis of sarcopenia plus NLR, PLR, and LMR

Neither systemic inflammation index alone was a significant predictor in multivariate analysis, but we also evaluated whether their utility could be improved by combining them with sarcopenia. We created ROC curves and compared the AUC values to assess the discrimination ability of each prognostic score. The AUC values of the sarcopenia alone, sarcopenia plus NLR, sarcopenia plus PLR, and sarcopenia plus LMR for OS were 0.678 (95%CI = 0.570–0.786), 0.756 (95%CI = 0.662–0.850), 0.727 (95%CI = 0.624–0.829), and 0.752 (95%CI = 0.655–0.848), respectively. Although the combination of sarcopenia and NLR, PLR or LMR showed significantly greater AUC values than sarcopenia alone, there were no significant differences between sarcopenia plus NLR, sarcopenia plus PLR and sarcopenia plus LMR.

### The effects of sarcopenia and systemic inflammation indices on survival

**Figure 3:**
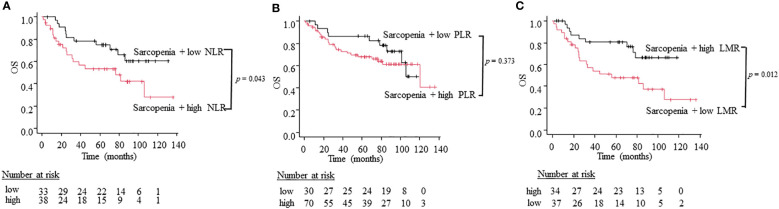
Kaplan-Meier curves comparing OS between the sarcopenia plus high inflammation status and low inflammation status defined by NLR **(A)**, PLR **(B)**, and LMR **(C)**.

**Figure 4:**
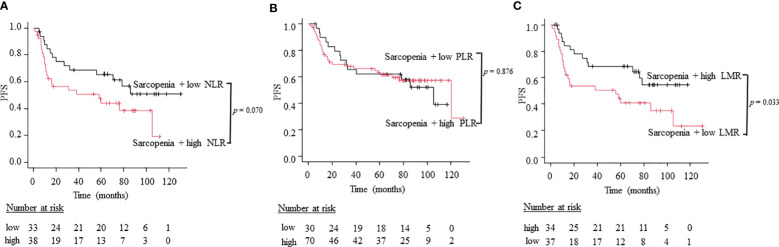
Kaplan-Meier curves comparing PFS between the sarcopenia plus high inflammation status and low inflammation status defined by NLR **(A)**, PLR **(B)**, and LMR **(C)**.

To compare the utility of three different combination patterns of sarcopenia and systemic inflammatory indices, we stratified patients into sarcopenia plus high inflammatory status and sarcopenia plus low inflammatory status using NLR, PLR, and LMR. Patients with sarcopenia plus high NLR had a worse OS than patients with sarcopenia plus low NLR (5-year OS; 66.7% vs. 39.4%, log-rank test: p = 0.043; **Figure 3A**). Similarly, patients with sarcopenia plus low LMR had significantly worse OS than those with sarcopenia plus high LMR (5-year OS; 67.6% vs. 37.8%, log-rank test: p = 0.012; **Figure 3C**). However, the combined index of sarcopenia and PLR did not show significant differences between two groups (5-year OS; 80.0% vs. 55.7%, log-rank test: p = 0.377; **Figure 3B**). On the other hand, regarding PFS, the combination of sarcopenia and LMR is the only indicator that showed significant differences between two groups (5-year PFS; 61.8% vs 32.4%, log-rank test: p = 0.033; **Figure 4C**), while sarcopenia plus NLR (5-year PFS; 60. 6% vs. 34.2%, log-rank test: p = 0.070; **Figure 4A**) and sarcopenia plus PLR (5-year PFS; 60.0% vs. 52.9%, log-rank test: p = 0.876; **Figure 4B**) showed no statistical differences, suggesting that the combination of sarcopenia and LMR is the most reliable prognostic index. We thus used this parameter in the subsequent subgroup analysis. The group of patients with sarcopenia plus low LMR had a higher percentage of oro-hypopharyngeal cancer (51.4% vs. 38.2%) and Stage III-IV cancers (62.2% vs. 44.1%) compared to those with sarcopenia plus high LMR. As for the treatment, the group of patients with sarcopenia plus low LMR tended to perform surgery compared to those with sarcopenia plus high LMR (37.8% vs. 20.6%), but there were no significant differences concerning treatment choice.

We then performed a multivariate analysis including sarcopenia plus LMR status, T classification, and albumin which showed significant differences in univariate analysis. The results revealed that sarcopenia plus low LMR was a significantly poor prognostic factor both for OS and PFS with greater HR values than sarcopenia alone (**Table 3**).

**Table 3:** Prognostic factor for OS and PFS in the multivariate analysis using the combined index of sarcopenia and LMR.

variables		OS		PFS	
		HR (95% CI)	*p*-value	HR (95% CI)	*p*-value
Sarcopenia + LMR status	Non–sarcopenia	1		1	
	Sarcopenia alone	3.97 (1.38–11.44)	0.011	2.48 (1.09–5.66)	0.031
	Sarcopenia + high LMR	2.43 (0.74–7.92)	0.142	1.84 (0.73–4.65)	0.196
	Sarcopenia + low LMR	5.46 (1.82–16.37)	0.002	3.08 (1.29–7.38)	0.011

OS (overall survival); PFS (progression-free survival); HR (hazard ratio); CI (confidence interval); LMR (lymphocyte/monocyte ratio).

## Discussion

Sarcopenia is reportedly highly associated with poor treatment outcomes in various cancer types, including HNSCC (4, 13, 14). Several studies demonstrated that sarcopenia correlates to increased surgical complications, such as delayed surgical wound healing (15), increased incidence of pharyngocutaneous fistula (15), surgical site infection (16), and postoperative delirium (17). Other studies reported that sarcopenia is associated with chemoradiation-induced toxicities in patients with HNSCC, as follows: mucositis, dysphagia, and dose-limiting chemotherapeutic toxicities (13). These side effects prevent the completion of full chemotherapeutic or radiation treatment cycles and lead to poor treatment outcomes. Moreover, several studies described that sarcopenia is an independent poor prognostic factor for OS and PFS in patients with HNSCC (14, 18). In our study, HNSCC patients with sarcopenia displayed poorer OS and PFS than those without sarcopenia. In addition, sarcopenia accompanied by systemic inflammation was closely associated with poor OS and PFS, and it was considered a more sensitive indicator than sarcopenia alone.

Systemic inflammation is intimately involved in tumor development, invasion, and metastasis (19). Moreover, several inflammatory markers, including NLR, PLR, and LMR, reportedly correlated with clinical outcomes in patients with HNSCC. Neutrophils release various inflammatory mediators that affect tumor angiogenesis, reduce T lymphocyte function, and promote tumor cell growth and metastasis (20–22). Lymphocytes also release several factors, that inhibit antitumor immunity and promote tumor growth and metastasis (23, 24), leading to an altered tumor microenvironment. Moreover, increased lymphocyte infiltration in the tumor microenvironment was reportedly associated with a better response to cytotoxic treatment and prognosis in patients with cancer (25). Platelets and monocytes promote tumor progression (26, 27). Platelets are activated in tumor cells and release several cytokines, thereby promoting tumor proliferation, metastatic potential, and angiogenesis (26). Monocytes infiltrate into the tumor cells, promote tumor progression and invasion, and suppress immune cell function (27). Several studies reported that higher NLR and PLR as well as lower LMR were independent poor prognostic factors in patients with HNSCC (6–8, 28, 29). However, we could not identify both NLR alone and LMR alone as an independent prognostic factor in our study. Multiple studies have described that systemic inflammation closely correlates to sarcopenia. Systemic inflammation could promote muscle catabolism through pro-inflammatory cytokines such as interleukin-6, tumor necrosis factor-alpha, and transforming growth factor-beta (30, 31). Furthermore, muscle breakdown might further exacerbate the existing systemic inflammation (32), resulting in a detrimental inflammation-myopia cycle (32). Higher NLR and PLR as well as lower LMR reportedly correlated with a higher sarcopenia incidence (7, 8, 33, 34). However, only a few studies have explored the relationship between systemic inflammation markers (NLR, PLR, and LMR) and sarcopenia in patients with HNSCC. In our study, we demonstrated that higher NLR and PLR as well as lower LMR were significantly associated with sarcopenia.

NLR, PLR, and LMR are reportedly poor prognostic factors in patients with HNSCC, although all are single prognostic factors. Recently, the combination of sarcopenia and systemic inflammation markers reportedly improved prognosis accuracy. Sarcopenia accompanied by systemic inflammation affects the prognosis in patients with various cancers. However, only a few studies have evaluated the efficacy of combining sarcopenia and inflammation on the prognosis of patients with HNSCC. Yamahara et al. (34) described that sarcopenia accompanied by high PLR was the most significant independent risk factor for OS and DFS. Cho et al. (35) reported that sarcopenia accompanied by high NLR was the most significant risk factor of poor OS and PFS, reflecting a very aggressive status in patients with HNSCC. Moreover, several studies described that sarcopenia plus lower LMR was an independent poor prognostic factor in various cancers (6–8). However, all these studies have examined single combination patterns of sarcopenia and systemic inflammatory markers (NLR, PLR, and LMR). In our study, we evaluated multiple combination patterns using comparative assessment and revealed that sarcopenia plus low LMR is a more perceptive indicator of poor prognosis than sarcopenia alone. As no studies have compared the efficacy of different combinations of indicators, our findings provide novel scientific contributions to cancer treatment. For high-risk patients detected by the combined index of sarcopenia and LMR, it may be useful to consider supportive therapy such as nutritional intervention with close monitoring. Immunonutrition is emerging as a promoting intervention that can attenuate sarcopenia-related inflammation to improve outcomes (36). They contain unique ingredients, such as arginine, omega-3 fatty acids, and dietary nucleotides that modulate prostaglandin E2 production, decrease IL-6 production, and promote T-cell differentiation (37). The previous paper reported that the use of immunonutrition for five days before surgery was associated with a significant reduction in the incidence of wound abscesses and orocutaneous or pharyngocutaneous fistulas compared to the control group (38), which may affect prognosis by allowing transition to appropriate adjuvant postoperative therapy.

Our study has some limitations. First, inevitable bias might be present in a single-center retrospective study related to sample size. Second, the CSA estimation method at L3 based on the CSA at C3 is uncertain. Jung et al. (39) reported another predictive model for estimating the CSA at L3 different from the approach of Swartz et al. (11). Moreover, they demonstrated that CSA at C3 alone displayed high predictability for estimating OS after definitive treatment for patients with advanced-stage HNSCC (39), leading to making the conversion from CSA at C3 to L3 unnecessary. Third, in previous studies, no consensus has been reached on the cut-off for sarcopenia, making result comparison difficult. Further studies with increased sample sizes would be required to support our findings.

## Conclusion

In this study, we described that sarcopenia accompanied by low LMR significantly correlated with poor OS and PFS in patients with HNSCC undergoing curative therapy. The combination of these two measures might be beneficial for identifying patients with HNSCC at risk of poor outcomes.

## Data availability statement

The original contributions presented in the study are included in the article/supplementary material. Further inquiries can be directed to the corresponding author.

## Ethics statement

The studies were approved by Research ethics committee of Saiseikai Utsunomiya Hospital. The studies were conducted in accordance with the local legislation and institutional requirements. The requirement for informed consent was waived owing to the retrospective nature of the analysis.

## Author contributions

KK: Conceptualization, Writing – review & editing, Data curation, Formal Analysis, Writing – original draft. TK: Conceptualization, Writing – review & editing, Investigation, Methodology, Project administration, Supervision, Validation. YS: Conceptualization, Investigation, Methodology, Project administration, Supervision, Validation, Writing – review & editing. MU: Conceptualization, Investigation, Methodology, Supervision, Writing – review & editing. HS: Conceptualization, Investigation, Methodology, Supervision, Writing – review & editing. YF: Conceptualization, Investigation, Methodology, Supervision, Writing – review & editing. SS: Conceptualization, Investigation, Methodology, Supervision, Writing – review & editing. HO: Conceptualization, Investigation, Methodology, Supervision, Writing – review & editing.
